# Hyperspectral Imaging-Based Deep Learning Method for Detecting Quarantine Diseases in Apples

**DOI:** 10.3390/foods14183246

**Published:** 2025-09-18

**Authors:** Hang Zhang, Naibo Ye, Jingru Gong, Huajie Xue, Peihao Wang, Binbin Jiao, Liping Yin, Xi Qiao

**Affiliations:** 1 College of Computer and Information Engineering, Tianjin Agricultural University, Tianjin 300384, China; zhanghrz@126.com (H.Z.); czsjunzi@126.com (P.W.); 2 Shenzhen Branch, Guangdong Laboratory for Lingnan Modern Agriculture, Genome Analysis Laboratory of the Ministry of Agriculture and Rural Affairs, Agricultural Genomics Institute at Shenzhen, Chinese Academy of Agricultural Sciences, Shenzhen 518120, China; yenaibo@outlook.com; 3 Technical Centre for Animal, Plant, and Food Inspection and Quarantine, Shanghai Customs, Shanghai 200002, China; kalen_17@163.com (J.G.); llgod@126.com (H.X.); jiaobinbin@shcc.edu.cn (B.J.); yinliping@hotmail.com (L.Y.)

**Keywords:** apple disease detection, import-export quarantine management, hyperspectral imaging, deep learning

## Abstract

Rapid detection of quarantine diseases in apples is essential for import–export control but remains difficult because routine inspections rely on manual visual checks that limit automation at port scale. A fast, non-destructive system suitable for deployment at customs is therefore needed. In this study, three common apple quarantine pathogens were targeted using hyperspectral images acquired by a close-range hyperspectral camera and analyzed with a convolutional neural network (CNN). Symptoms of these diseases often appear similar in RGB images, making reliable differentiation difficult. Reflectance from 400 to 1000 nm was recorded to provide richer spectral detail for separating subtle disease signatures. To quantify stage-dependent differences, average reflectance curves were extracted for apples infected by each pathogen at early, middle, and late lesion stages. A CNN tailored to hyperspectral inputs, termed HSC-Resnet, was designed with an increased number of convolutional channels to accommodate the broad spectral dimension and with channel and spatial attention integrated to highlight informative bands and regions. HSC-Resnet achieved a precision of 95.51%, indicating strong potential for fast, accurate, and non-destructive detection of apple quarantine diseases in import–export management.

## 1. Introduction

Apples are among the most widely cultivated and consumed fruits worldwide. Global production reached 87 million metric tons in 2019 [1]. Shifts in consumer preferences toward higher quality and variety have driven greater demand for imported fruit, particularly apples. In China, the value of apple imports increased from USD 181.38 million in 1997 to USD 688.5 million in 2022 [2].

In parallel, problems associated with fungal infection have become more prominent [3]. During transport, apples are frequently affected by fungal diseases, leading to quality degradation and, in severe cases, spoilage. These outcomes cause substantial economic losses and undermine market reputation, reducing consumers’ willingness to purchase imported apples. Quarantine fungal pathogens are of particular concern because they threaten not only fruit quality but also agricultural biosecurity in importing countries [4].

Detection of quarantine pathogens in imported apples has primarily relied on traditional biological assays such as polymerase chain reaction (PCR) and DNA sequencing [5]. These techniques are destructive to samples, labor-intensive, and time-consuming, rendering them unsuitable for rapid inspection at ports of entry. Infections caused by *Cytospora mali*, *Monilinia fructigena*, and *Botryosphaeria dothidea* typically result in fruit decay that begins as surface lesions. As infection advances, lesions darken and expand, and the fruit progressively softens. Because symptom trajectories are highly similar across these pathogens, and because quarantine regulations often restrict available sample sizes, the development of accurate and generalizable identification algorithms is significantly constrained.

Hyperspectral imaging is a technique that integrates spatial and spectral information [6] and offers clear advantages over conventional RGB imaging. Reflectance imaging spectroscopy was employed, capturing hundreds of narrow bands across the visible and near-infrared, each providing distinct spectral cues. This rich spectral content enables discrimination among samples with very similar phenotypes, particularly when sample availability is limited. In recent years, hyperspectral imaging has been widely adopted in agriculture [7], food science [8], and remote sensing [9]. With continuing advances in sensors and data-processing algorithms, hyperspectral imaging has become a powerful tool, improving the precision and efficiency of crop phenotyping, plant disease detection, and quality assessment of agricultural products.

Recent research efforts have increasingly focused on near-range hyperspectral imaging technology for analyzing crop phenotypic characteristics [10]. This study developed a peach damage–detection method based on gray-level co-occurrence matrix (GLCM) features. Texture descriptors extracted with GLCM were used to train Random Forest (RF) and Extreme Gradient Boosting (XGBoost) models for prediction. However, the work evaluated only classical machine-learning approaches and did not compare against convolutional neural networks (CNNs) or other deep-learning methods.

With rapid advancements in hyperspectral imaging and artificial intelligence (AI), the fusion of these two technologies provides novel opportunities for plant disease and pest detection. Hyperspectral imaging leverages variations in reflection, absorption, and scattering properties of different materials to capture comprehensive spectral and spatial image information. Meanwhile, CNNs have significantly advanced the field of image recognition. A 2D/3D hybrid CNN incorporating an Feature Pyramid Network (FPN) and hybrid attention was developed for classifying hyperspectral images [11]. Another study proposed an improved Vision Transformer (ViT) for crop disease recognition that leverages an AFNO-based Transformer to pre-extract global features, markedly improving leaf-disease identification in complex natural environments [12]. The integration of hyperspectral imaging with deep learning methods allows advanced image-recognition algorithms to fully exploit hyperspectral data, extracting highly valuable features. This combination substantially expands the dimensionality and richness of data available for computer-vision tasks compared to traditional RGB imagery, enhancing classification accuracy and processing efficiency in practical scenarios.

Although hyperspectral imaging has been shown to extract phenotypic information effectively and CNN have demonstrated reliable performance in disease and pest image classification, CNN-based classification of hyperspectral images of apple diseases remains underexplored. To address the slow throughput and heavy labor demands encountered in quarantine inspection under real import and export conditions, an integrated hyperspectral–CNN approach was developed. A field-deployable automated device for hyperspectral detection of apple diseases was designed for use at inspection sites. The system improves detection efficiency and reduces operational costs.

**Figure 1:**
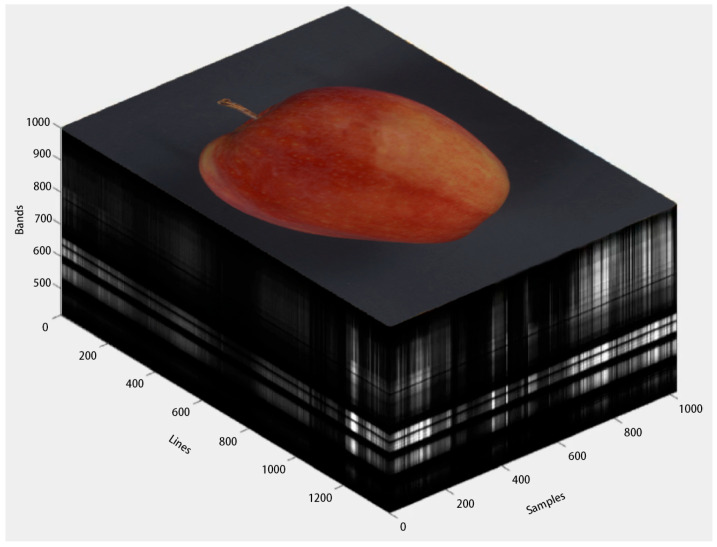
Stereo view of hyperspectral data. The image’s lines, samples, and bands form a cube-like three-dimensional structure.

In this study, apple quarantine diseases were examined by capturing hyperspectral images of apples infected by *Cytospora mali*, *Monilinia fructigena*, and *Botryosphaeria dothidea*. The hyperspectral camera captured wavelengths from 400 to 1000 nm, and the structure of the resulting image cube is shown in Figure 1. Changes in spectral curves were analyzed across early, middle, and late stages of infection. Dimensionality reduction with Competitive Adaptive Reweighted Sampling (CARS) was evaluated and reduced the original 448 bands to 76 informative bands. Hyperspectral imaging was then combined with convolutional neural network–based recognition, and a model named HSC-ResNet was proposed for hyperspectral classification. The architecture was adapted to accommodate the large spectral input and incorporates channel and spatial attention mechanisms to emphasize salient features. With this design, high efficiency and accuracy were achieved for detecting quarantine diseases in apples.

## 2. Materials and Methods

### 2.1. Sample Collection and Preparation

‘Gala’ apples (*Malus domestica* ‘Gala’) imported from New Zealand were used as experimental material. Prior to sampling, all fruit passed import quarantine to verify freedom from regulated pests and diseases in accordance with the International Standards for Phytosanitary Measures (ISPM) [13]. Fruit with mechanical injury, visible infection, or other surface defects were excluded to meet experimental quality criteria. The remaining apples were divided into five groups: fruit inoculated with *Botryosphaeria dothidea*, *Monilinia fructicola*, or *Cytospora mali*, and two control groups comprising healthy fruit and mechanically injured fruit.

### 2.2. Pathogen Identification by PCR Technique

Fungal pathogens were inoculated into apple samples using a mycelium-block method [14]. Mycelial blocks (5 mm diameter) were excised from the actively growing colony margin on culture plates with a sterile biopsy punch. The inoculation site on each fruit was surface-disinfected with 70% ethanol. A 5 mm core of peel and underlying flesh was then removed with the same punch, and the mycelial block was placed into the cavity with the mycelium oriented inward. Inoculated apples were sealed in plastic containers and incubated at 22 °C under humid conditions. Uninoculated apples handled in parallel served as negative controls.

PCR assays were conducted on infected apples to prevent contamination by unintended fungi [15]. Pathogen DNA was rapidly extracted directly from infected apple tissues using the alkaline lysis method [16], infected tissue was placed into a 2-mL centrifuge tube, to which 50 µL of 0.5 mol L^−1^ NaOH was added. After thorough shaking and centrifugation, 10 µL of the resulting supernatant was transferred into 90 µL of 100 mmol L^−1^ Tris buffer (pH 8.0), providing crude DNA extract suitable for PCR amplification. The extracted DNA could either be used immediately for PCR amplification or stored at −20 °C until use.

Specific primer pairs were utilized to identify *Botryosphaeria dothidea*, *Monilinia fructicola*, and *Cytospora mali*, respectively. Primer sequences and expected PCR product sizes are presented in Table 1. Each PCR reaction had a total volume of 50 µL, containing 1 × PCR buffer without Mg^2+^, 1.5 mmol L^−1^ MgCl_2_, 0.2 mmol L^−1^ of each dNTP, 0.2 µmol L^−1^ each of forward and reverse primers, 1.25 U of Taq DNA polymerase, and 2 µL template DNA. The volume was adjusted to 50 µL with ddH_2_O. PCR amplification conditions consisted of initial denaturation at 94 °C for 3 min, followed by 30 cycles of denaturation at 94 °C for 30 s, annealing at 55 °C for 30 s, and extension at 72 °C for 90 s. A final extension step was carried out at 72 °C for 10 min before termination.

**Figure 2:**
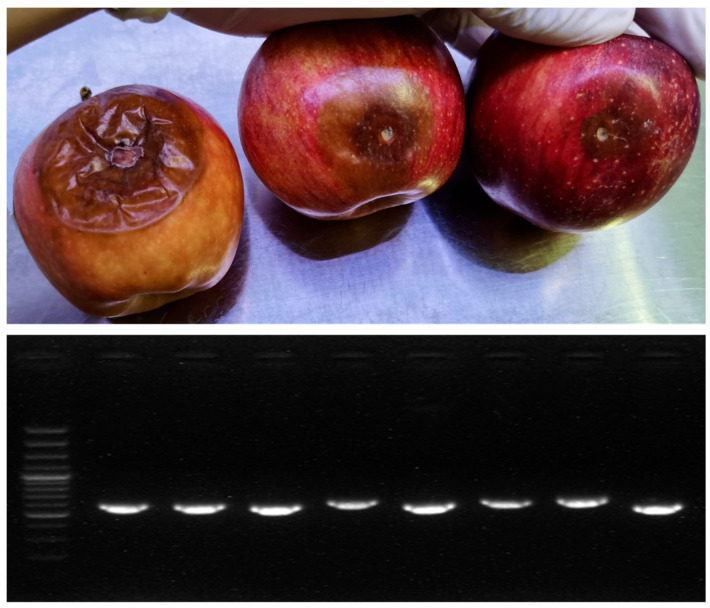
Representative images of inoculated apple samples, along with PCR products visualized using a gel imaging system, are presented.

Following PCR amplification, products were resolved on a 1.5% agarose gel, visualized with a gel imaging system, and their quality assessed, as shown in Figure 2. Additionally, specific primers targeting the internal transcribed spacer (ITS) regions of fungal DNA were used for PCR amplification. The obtained sequences were compared with available sequences in the NCBI database to confirm the accuracy of the pathogen identification.

### 2.3. Hyperspectral Imaging System

**Figure 3:**
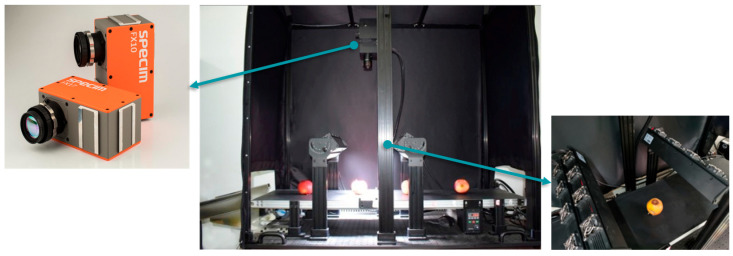
Mechanical structure.

A hyperspectral camera was mounted directly above a conveyor belt, the camera used was the Specim FX10, designed by SPECIM (Finland). Uniform illumination was provided to maintain consistent lighting across apple surfaces. Shadows and specular reflections that could degrade image quality were thereby minimized. The mechanical structure is shown in Figure 3. As apples traveled along the belt, the camera recorded spectral data continuously at a high frame rate across 400–1000 nm. Detailed specifications of the hyperspectral camera are listed in Table 2.

### 2.4. Image Preprocessing

#### 2.4.1. Average Spectral Extraction

**Figure 4:**
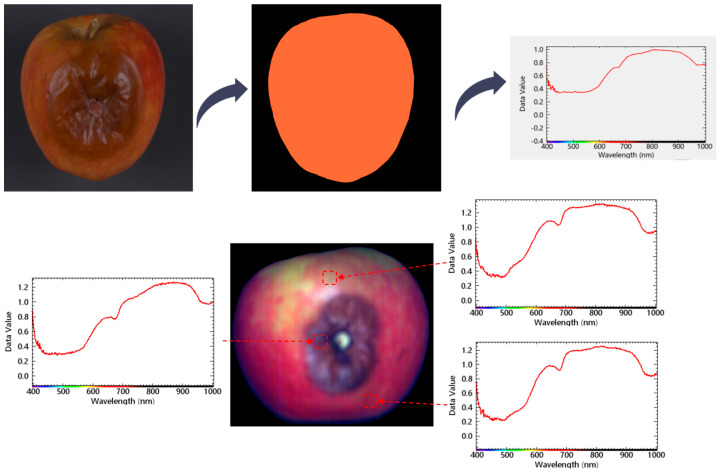
Process of Extracting Average Spectrum from Apples.

Obtaining stable and representative spectral characteristics is crucial for accurate modeling and disease identification from hyperspectral imagery. However, practical spectral analysis often encounters challenges from background interference, shadows, and non-target regions [17]. Extracting spectra without addressing these factors reduces robustness and degrades accuracy. All hyperspectral images were first calibrated using black and white references [18]. Annotation masks were then applied to exclude background, and mean reflectance spectra were computed from apple regions for subsequent analysis. The procedure is illustrated in Figure 4.

**Figure 5:**
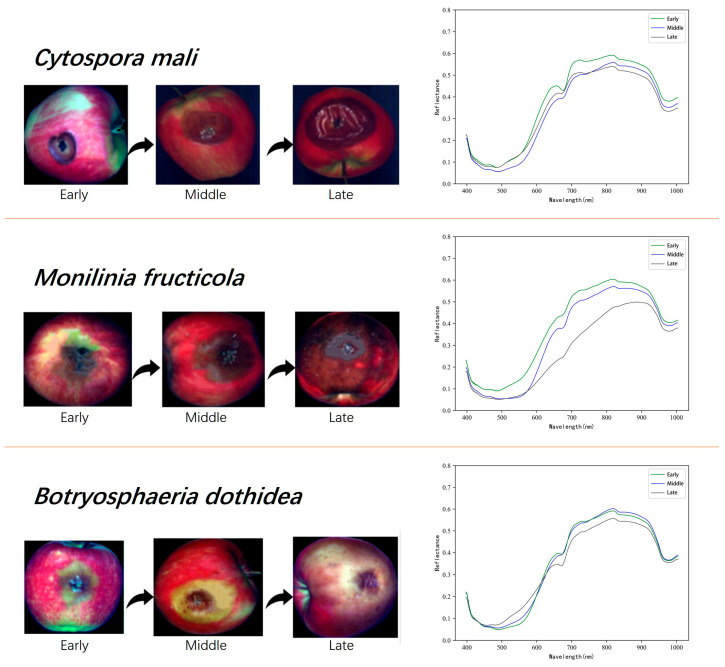
Example images of apples at early, middle, and late infection stages, together with the corresponding mean spectral curves derived from spectra extracted across all samples.

Average spectra were computed as the mean reflectance of apple samples at each wavelength, yielding representative spectral profiles. Variability in individual spectra can arise from sample condition, viewing angle, and illumination. Averaging mitigates this variability, smooths individual differences, and reduces the influence of outliers. The resulting profiles emphasize overall spectral characteristics and support more robust downstream analysis. Features derived from inter-class spectral differences in the averaged profiles help suppress noise and improve the generalization of classification, regression, and predictive models. Representative images of early, middle, and late infection stages for the three pathogens, together with a comparison of their average spectral curves, are presented in Figure 5.

The hyperspectral characteristics of apples infected with *Cytospora mali*, *Monilinia fructicola*, and *Botryosphaeria dothidea* were examined across early, middle, and late stages of infection. Distinct visual trajectories were observed. *Cytospora mali* initially presented as localized epidermal necrosis that extended deeper into the fruit over time, culminating in visible mold layers. *Monilinia fructicola* showed mild symptoms at first but later developed large necrotic regions characterized by tissue drying and darkening. *Botryosphaeria dothidea* began with localized pigment deposition and progressed to extensive tissue liquefaction and structural collapse.

Spectrally, the three diseases displayed similar overall reflectance patterns. Reflectance in the 400–500 nm (blue) region remained low, consistent with absorption by chlorophyll and carotenoids. Within the visible range (400–700 nm), higher reflectance was observed at early stages, indicating limited pigment degradation. In the near-infrared range (700–1000 nm), reflectance declined as infection advanced, reflecting water loss and structural damage in the tissue.

Stage-specific differences were also apparent: *Cytospora mali* showed the largest spectral changes between 700–900 nm, *Monilinia fructicola* displayed maximal variation across 600–900 nm, and *Botryosphaeria dothidea* exhibited distinct changes primarily at 500–600 nm and 800–900 nm. Because simple metrics could not reliably quantify and distinguish the pathogens’ spectral signatures, the study subsequently employed AI-based feature selection to identify key hyperspectral bands, thereby enabling effective dimensionality reduction.

#### 2.4.2. Dimensionality Reduction

Previous analyses indicated that not every wavelength contributes meaningfully to classification. The high dimensionality of hyperspectral data complicates downstream analysis and modeling. Reducing the number of bands removes redundancy, decreases data volume, and lowers computational demand while retaining the most informative features for classification or regression, thereby improving accuracy and stability.

To address this challenge, Competitive Adaptive Reweighted Sampling (CARS) was applied to select an informative subset of spectral bands and discard noise [19,20]. This procedure reduces dimensionality, often maintains or improves model accuracy, and markedly shortens computation time while increasing robustness. CARS is widely used in spectral analysis and chemometrics and is particularly effective for high-dimensional datasets such as hyperspectral imagery.

In competitive screening of high-dimensional features like spectral bands, the process begins by calculating regression coefficients for each feature using a model such as partial least squares (PLS) [21]. The absolute values of these coefficients are then normalized to obtain feature weights.

(1) $$w_{j}=\frac{\left|\beta_{j}\right|}{\sum_{i=1}^{m}\left|\beta_{i}\right|},\left(j=1,\;2,\ldots ,m\right)$$

Here, $\beta_{j}$ denotes the regression coefficient of the j-th feature estimated by the PLS model, and ${\left\{\beta_{i}\right\}}_{\mathrm{i}=1}^{m}$ denotes the set of regression coefficients for all m features obtained from the PLS model. These coefficients quantify the relative influence of each feature on the response variable, and their absolute values are normalized to yield the weights $w_{j}$. A larger $w_{j}$ indicates a greater contribution of that feature to the model.

Subsequently, to shrink the feature set step by step, CARS applies an exponential-decay function in the k-th iteration to determine the retention ratio for that round and, from this, calculates how many features to keep.

(2) $$r_{k}=r_{max}{\left(\frac{r_{min}}{r_{max}}\right)}^{\frac{k-1}{K-1}}$$

(3) $$mₖ=\lfloor m\times r_{k}\rfloor$$

Building on this, a competitive mechanism—implemented through ranking or random sampling—retains the top mₖ features with the highest weights and removes those with lower weights. After multiple iterations, the subset that delivers the best performance on the validation set is chosen, achieving dimensionality reduction while improving model accuracy.

Applying CARS reduced the number of bands from 448 to 76. The reduction decreases data volume and computational load while preserving informative wavelengths, thereby accelerating training and recognition. Nevertheless, approaches based solely on traditional machine learning show limited performance on hyperspectral datasets [22]. In subsequent experiments, PLS using the 76-band subset achieved 61.89% accuracy, whereas the best single-band model reached 41.02%, indicating that CARS alone yields limited accuracy.

To enhance classification accuracy, convolutional neural networks for hyperspectral image classification were further investigated using the selected bands.

### 2.5. Deep Learning Models

Rapid advances in artificial intelligence have produced numerous convolutional neural network architectures [23], each with unique strengths. In this study, five representative models—ShuffleNet [24], MobileNet [25], EfficientNet [26], ViT [27], and ResNet [28]—were trained on the hyperspectral dataset. These architectures differ in structural design, computational efficiency, generalization capability, and scalability. ShuffleNet and MobileNet support lightweight deployment on mobile devices. EfficientNet improves performance through compound scaling. ViT introduces Transformer-based vision modeling beyond purely convolutional designs. ResNet remains a foundational deep CNN architecture. Because these models are widely used and each offers distinct strengths, they were selected for comparison on the apple-disease hyperspectral dataset.

We compared recognition accuracy and parameter counts across the networks. ResNet achieved the highest accuracy on hyperspectral images at 87.78%. ShuffleNet used the fewest parameters at 5.42 million. Detailed results are shown in Table 3. However, despite their effectiveness in conventional image tasks, these standard models face challenges in handling the high-dimensional spectral data typical of hyperspectral imagery.

Among the compared architectures, ResNet’s residual topology—identity shortcuts with layer-wise feature reuse—improves optimization stability and gradient flow, alleviates degradation, and enables deeper, more robust representations on high-dimensional, redundant, and noisy hyperspectral data. Its bottleneck units employ 1 × 1 convolutions for cross-channel linear combination and 3 × 3 convolutions to model local spatial–spectral couplings, thereby enhancing channel interaction and effective receptive field while controlling parameters and computation. By contrast, the depthwise separable convolutions and channel grouping in ShuffleNet and MobileNet substantially weaken cross-channel modeling, making subtle inter-band differences harder to capture; ViT is more sensitive to data scale and regularization and is not advantageous at the present dataset size; EfficientNet benefits from compound scaling, yet its MBConv blocks remain dominated by per-channel operations with limited cross-channel coupling. These architectural distinctions are consistent with the empirical results: on this hyperspectral dataset, ResNet achieved the highest accuracy (87.78%), and its residual, modular design readily accommodates hyperspectral-specific operators such as spectral attention.

Given ResNet’s superior performance on the apple disease dataset, it was adopted as the baseline for further optimization, yielding HSC-ResNet, a variant specifically adapted for hyperspectral image classification.

#### 2.5.1. HSC-ResNet Model

HSC-ResNet was designed specifically for high-throughput hyperspectral image classification. CNNs are optimized for RGB images with only three spectral channels, whereas the images in this study contain 448 bands. Such high-dimensional input can overwhelm standard architectures, so ResNet50 was restructured to handle hyperspectral data more effectively.

The revised network retains the residual learning strategy of ResNet50. This design helps mitigate gradient vanishing and performance degradation in deep architectures. The input channel dimension is expanded from 3 to 76. These 76 channels are the informative bands selected from the original 448 by CARS. The wider input accelerates both training and inference. Channel and spatial attention are incorporated through the Convolutional Block Attention Module (CBAM), enabling the network to emphasize the most informative spectral channels and spatial regions.

**Figure 6:**
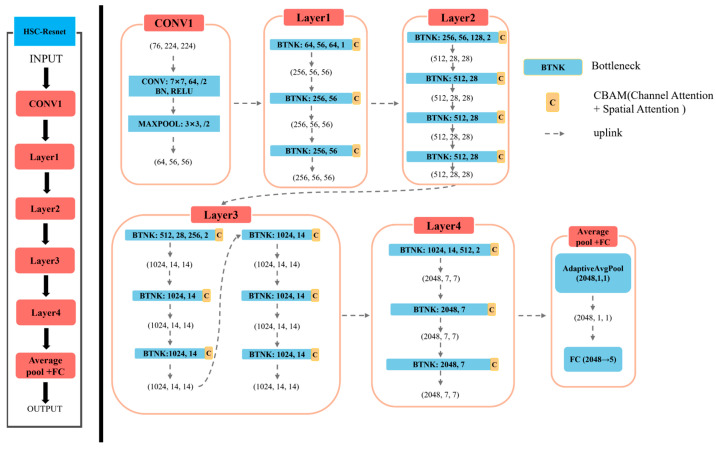
HSC-Resnet Model Architecture Diagram.

The input tensor, shaped (batch, 76, 224, 224), first passes through a convolution–batch normalization–ReLU sequence that extracts low-level spatial–spectral features and reduces the spatial resolution to 112 × 112. Max pooling further reduces the feature map to 56 × 56, lowering computational cost and mitigating overfitting. Feature abstraction proceeds through four residual stages, producing a tensor of size (batch, 2048, 7, 7). After each residual block, the CBAM module reinforces the channels and spatial areas most relevant for classification. Adaptive average pooling then condenses each feature map to a single value, generating a (batch, 2048, 1, 1) tensor. Finally, a fully connected layer maps these high-level descriptors to the target classes, completing the classification. The detailed model architecture is shown in Figure 6.

In total, the architecture includes fifty convolutional layers, excluding pooling operations and the final dense layer. With its adapted channel configuration, integrated attention mechanisms, and preserved residual connections, HSC-ResNet is well suited to the high dimensionality of hyperspectral imagery while remaining computationally efficient.

#### 2.5.2. CBAM Attention Mechanism

Hyperspectral images are inherently high dimensional. Representing many wavelengths requires a large number of channels. This high channel count makes it difficult for a network to capture target features effectively. To mitigate this limitation, an attention module was adopted as a key optimization. The Convolutional Block Attention Module (CBAM), proposed in 2018, integrates channel and spatial attention [29]. Because hyperspectral data contain far more spectral bands than standard RGB images, identifying informative features is more challenging. In CBAM, the channel-attention branch assigns greater weight to the most discriminative spectral channels while suppressing redundant ones, and the spatial-attention branch emphasizes the most relevant regions within each feature map. Together, these mechanisms guide the network toward the spectral channels and spatial locations most critical for classification, thereby enhancing discriminative capacity.

The channel attention branch estimates the importance of each channel in the input feature map and assigns a learned weight. It applies both global max pooling and global average pooling, followed by a shared multi-layer perceptron (MLP) to capture channel importance.

The spatial attention branch then evaluates the significance of each spatial position in the feature map, building on the output of channel attention. It performs average pooling and max pooling along the channel dimension to produce two spatial maps, concatenates them, and processes the result with a 7 × 7 convolution to integrate the information. A Sigmoid activation generates the final spatial attention map.

### 2.6. Experimental Setup

#### 2.6.1. Hardware and Software Setup

To ensure a fair comparison, all experiments were run under identical hardware and software conditions. All models were trained in PyTorch 2.3.0. The complete hardware and software specifications are provided in Table 4.

To accelerate training, all hyperspectral images underwent uniform black and white calibration, followed by data augmentation. A total of 1628 images were used, randomly divided into training, validation, and test sets in a 6:2:2 ratio. The training set was used for parameter learning, the validation set for monitoring generalization and convergence during training, and the test set—kept untouched until training was complete—served for the final evaluation on unseen samples.

#### 2.6.2. Training Configuration

Tests indicated that the following hyperparameters yielded stable and reliable performance. The network was trained for 100 epochs, balancing accuracy with computational efficiency. After each epoch, accuracy and loss were measured on the validation set to monitor convergence. The batch size was set to 32 to maximize GPU utilization. A learning rate scheduler reduced the rate by 0.8 every 10,000 steps, and twelve worker threads were used to preprocess and load data in parallel. Training began with an initial learning rate of 1 × 10^−4^ and employed the AdamW optimizer with cosine annealing for automatic adjustment [30]. Detailed hyperparameters settings are listed in Table 5.

#### 2.6.3. Evaluation Metrics

Model performance on the hyperspectral classification task was evaluated using a confusion matrix [31] and overall accuracy. In the confusion matrix, rows denote the true classes and columns denote the predicted classes. Correct predictions appear on the main diagonal, whereas off-diagonal entries indicate misclassifications and provide a class-wise view of separability. Overall accuracy, computed from the confusion matrix, represents the proportion of correct predictions across all classes and offers a concise summary of model effectiveness.

Table 6 presents the standard form of a confusion matrix for binary classification. Rows give the true class of each sample—the top row for true positives, the bottom row for true negatives—whereas columns list the model’s predictions, with the left column indicating a positive prediction and the right column a negative one. Within this layout, TP (true positive) counts samples that are actually positive and predicted as positive; FN (false negative) counts those that are positive but predicted as negative; FP (false positive) counts samples that are negative yet predicted as positive; and TN (true negative) counts those that are negative and predicted as negative. Together, these four values provide the basis for calculating overall accuracy [32].

(4) $$Accuracy=\frac{Correct\;Predictions}{Total\;Samples}=\frac{TP+TN}{TP+TN+FP+FN}$$

Accuracy reflects the overall correctness of a model’s predictions—the proportion of all samples it classifies correctly.

## 3. Results

### 3.1. Training Analysis

**Figure 7:**
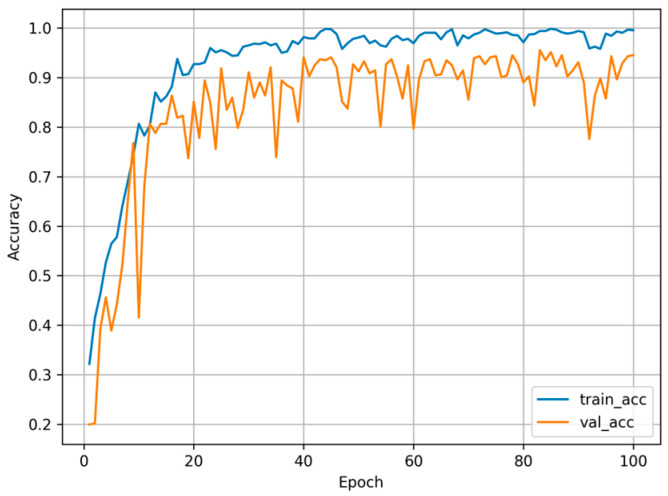
Training convergence curves of the HSC-ResNet model.

The HSC-ResNet model was trained and evaluated multiple times to verify its effectiveness in classifying hyperspectral images of apple diseases. Across these runs, it achieved an accuracy of 95.51%, demonstrating strong practical potential. The training convergence curves are shown in Figure 7. On the five-class task, the network exhibited robust feature extraction and discrimination. Among the evaluated models, HSC-ResNet attained the highest accuracy, suggesting that its architecture and integrated attention mechanisms enable more comprehensive use of hyperspectral information. Compared with related studies, a fruit recognition system based on a 3D CNN reported an accuracy of 90.47% [33], and a CNN trained on principal component images achieved 93.33% [34]. In comparison, the optimized HSC-ResNet reached 95.51% accuracy on the present dataset, indicating stronger practical utility.

The hyperspectral dataset used in this study contains 1628 apple images divided into five categories: *Cytospora mali*, *Monilinia fructicola*, *Botryosphaeria dothidea*, healthy fruit, and mechanically injured fruit. These classes reflect real inspection scenarios. *Cytospora mali*, *Monilinia fructicola*, and *Botryosphaeria dothidea* are common quarantine concerns in imported apples. They spread readily and can cause substantial economic loss. Healthy samples give the model a clear reference for non-diseased tissue. Mechanically injured samples are harder to distinguish because their spectra can partly resemble disease signals.

**Figure 8:**
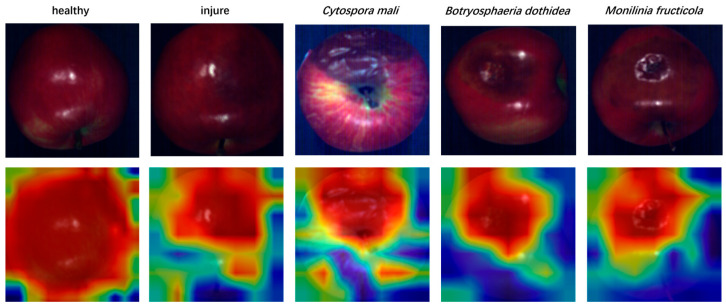
Attention Heatmap Comparison.

Classification performance across these five categories therefore offers a direct indication of the model’s suitability for practical industrial applications. To examine the network’s focus during inference, we visualized HSC-ResNet’s attention maps [35]. The heatmaps reveal that the model concentrates on regions surrounding lesions, an observation that supports further interpretability and refinement. Examples of attention heatmaps are shown in Figure 8. This visual evidence also highlights the benefit of the CBAM module: by combining channel and spatial attention, it directs the network to emphasize disease regions while suppressing background noise.

**Figure 9:**
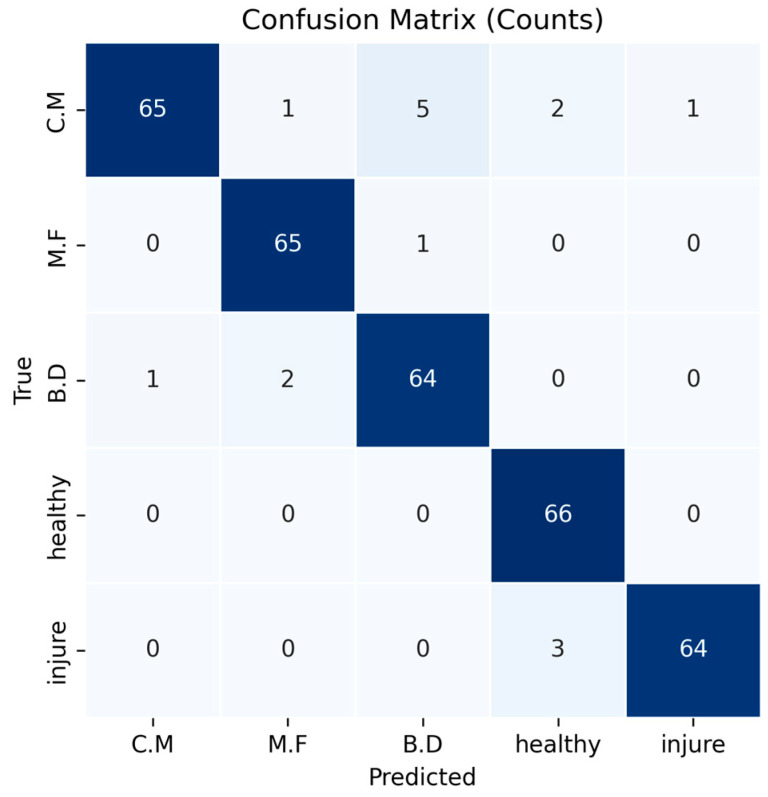
Confusion Matrix. In the figure, the class labels are C.M for *Cytospora mali*, M.F for *Monilinia fructicola*, and B.D for *Botryosphaeria dothidea*.

Visualizing the confusion matrix highlights where the classifier still encounters difficulties. Most disease categories are recognized with high precision, with *Monilinia fructicola* and *Botryosphaeria dothidea* showing particularly strong performance. However, accuracy drops for the “injury” class, where mechanically damaged fruit is sometimes misclassified as healthy. This overlap likely results from the spectral similarity between damaged and intact tissue, and shadows or background reflections can further obscure the distinction. Detailed confusion matrices for each class are shown in Figure 9.

**Figure 10:**
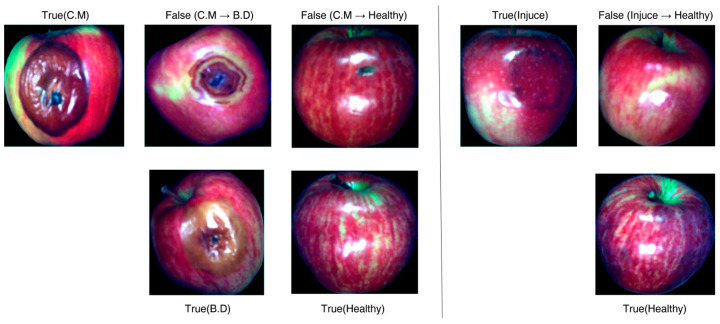
Comparison of misclassified and correctly classified samples.

Comparing the predicted labels with ground truth shows that most misclassifications occur in the *Cytospora mali* and injury classes. A closer look reveals two distinct patterns. First, several *Cytospora mali* samples are labelled as *Botryosphaeria dothidea* because lesions of the former share visual traits with the latter at certain developmental stages, misleading the network. Early *Cytospora mali* infections are also problematic: the lesion covers only a small fraction of the fruit surface, so the model often mistakes the sample for healthy fruit. Second, some injury samples are classified as healthy because the physical bruises are minor, making their spectral signatures resemble intact tissue and thus harder to detect. A comparison between misclassified and correctly classified samples is shown in Figure 10.

### 3.2. Ablation Study

To quantify the contribution of each design choice, ablation experiments were conducted on three factors: expansion of input channels, insertion of the CBAM attention module, and optimization of key training hyperparameters. Summary statistics are provided in Table 7.

The baseline, Model A, is a standard ResNet50 that accepts only three input channels. Hyperspectral bands are compressed into these three channels prior to training, resulting in an accuracy of 87.78%. While sufficient for tasks that do not require high precision, this approach discards most spectral information and fails to leverage the full potential of hyperspectral data.

In Model B, the input layer is expanded from 3 to 76 channels, enabling the network to utilize richer spectral detail. Accuracy increases to 92.13%, a gain of 4.35% points over the baseline. However, this wider input prevents the direct use of pre-trained ImageNet weights, requiring training from scratch and making convergence more challenging.

Model C builds on Model B by incorporating a CBAM module after each residual block. Channel attention is especially beneficial with seventy-six spectral bands, as it directs the network toward the most informative wavelengths, while spatial attention highlights relevant regions within each feature map. This combination boosts accuracy to 95.11%, an additional 2.98% improvement, providing strong evidence of CBAM’s value.

**Figure 11:**
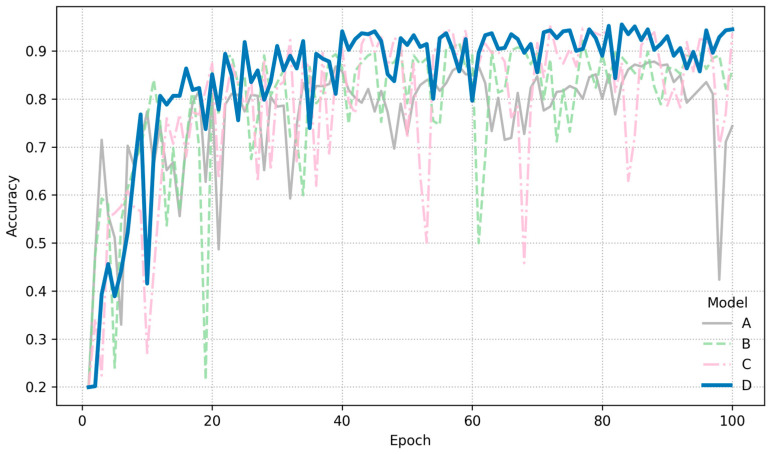
Convergence curve comparison of the ablation study.

Model D (HSC-ResNet) retained the expanded input and CBAM while refining the training strategy. Use of the AdamW optimizer with a cosine-annealing schedule reduced overfitting and stabilized gradients, producing the smoothest convergence among all configurations. Final accuracy reached 95.51%. The training convergence curves of these models are compared in Figure 11.

## 4. Discussion

This study began by extracting average spectral curves from hyperspectral images to examine spectral changes at early, middle, and late infection stages for each apple disease category. Specific hyperspectral wavelengths correspond to distinct biological traits—for instance, bands near 970 nm are closely linked to water content [36], while the 500–700 nm range reflects pigment variations associated with disease symptoms. On this basis, temporal evolution of spectral signatures was tracked throughout infection. Subtle inter-disease differences, however, were not readily distinguished by visual inspection of averaged spectra alone, which motivated the use of machine-learning-based spectral feature selection followed by deep-learning-based classification.

For spectral data processing, the CARS algorithm was employed to reduce dimensionality and redundancy, yielding 76 informative bands from the original 448. Although hyperspectral imagery is information rich, not every wavelength contributes equally to classification. Bands with minimal interclass variance can slow training, hinder convergence, and increase inference time. A natural question is whether one or a few highly informative wavelengths would suffice. In experiments conducted here, methods using only a small number of bands typically achieved accuracies below 60%. By contrast, the 76-band subset selected by CARS markedly outperformed single- or few-band inputs, underscoring the value of optimal band selection. In practice, identifying such a subset can enable more compact sensors, reduce acquisition requirements, and lower transmission and computation costs, thereby improving speed and operational efficiency.

Exclusive reliance on spectral feature selection has limitations. By reducing each hyperspectral image to a single spectral curve (wavelength on the x-axis, reflectance on the y-axis), CARS discards spatial context, which constitutes a substantial loss for hyperspectral analysis. To exploit both spectral and spatial cues, convolutional neural network (CNN) architectures capable of jointly modeling multiband spectral detail and spatial structure were therefore adopted.

Multiple established deep learning architectures were evaluated, including ShuffleNet, MobileNet, EfficientNet, ViT, and ResNet. ResNet produced the strongest performance for apple disease classification and was adopted as the baseline. The architecture was then extended to form HSC-ResNet, with the input expanded from three RGB channels to 76 hyperspectral bands to make fuller use of spectral and spatial information. Incorporation of the CBAM module further improved performance by directing channel attention to informative wavelengths and spatial attention to lesion-relevant regions. Hyperparameters were optimized to ensure stable convergence with high-dimensional input. The resulting HSC-ResNet achieved 95.51% accuracy on a challenging five-class dataset (*Cytospora mali*, *Monilinia fructicola*, *Botryosphaeria dothidea*, healthy samples, and mechanically injured fruit), meeting practical detection requirements.

Despite these promising outcomes, limitations remain. The dataset comprised only 1628 images, which is small by deep-learning standards. In addition, expansion of the input channels precluded the use of RGB-pretrained weights and made convergence more difficult. Together, these factors elevate the risk of overfitting and indicate a need for stronger regularization and more effective optimization strategies. Only a single cultivar was included: Gala apples imported from New Zealand. To better reflect the complexity of real import–export inspections, future studies should expand to more diverse and larger datasets.

Furthermore, the hyperspectral camera captured continuous spectra at 448 wavelengths spanning 400 to 1000 nm. During deep learning, only 76 bands were effectively used. Future work could optimize acquisition hardware to capture just these selected wavelengths at the source, greatly increasing imaging speed and detection efficiency.

## 5. Conclusions

This study addressed the urgent need for early detection and accurate identification of key quarantine apple diseases—*Cytospora mali*, *Monilinia fructicola*, and *Botryosphaeria dothidea*—that pose serious challenges in the apple import–export trade. By integrating hyperspectral imaging, which captures the optical reflection properties of both apple peel and sub-surface tissues, with advanced deep learning techniques, we enabled efficient feature extraction and accurate disease classification. This approach overcomes key limitations of traditional manual inspection and chemical testing, such as subjectivity, slow turnaround, and sample destruction.

For band selection and optimization, a comprehensive framework integrating spectral preprocessing, optimal wavelength selection, and neural network optimization was established to achieve robust plant-disease identification. Initial analyses of inter-disease spectral differences highlighted the need to balance accuracy with computational efficiency. Although hyperspectral imaging offers hundreds of informative bands, the resulting dimensionality imposes substantial computational cost. Comparative experiments showed that selecting 76 informative wavelengths from the original 448 reduced dimensionality, computational load, and training time without compromising accuracy.

For the design of the hyperspectral image recognition model, several established deep learning architectures were first evaluated. An optimized variant tailored for hyperspectral classification, HSC-ResNet, was then developed. The input channel dimensionality was expanded to accommodate hyperspectral data, the CBAM attention mechanism was integrated to emphasize informative wavelengths and lesion regions, and key hyperparameters were tuned to ensure stable convergence. On a five-class apple disease dataset, HSC-ResNet achieved 95.51% accuracy, satisfying the precision and reliability requirements of quarantine inspection.

In summary, the combination of hyperspectral imaging and deep learning presented here provides a practical pathway for rapid, non-destructive, and highly accurate identification of major quarantine apple diseases. The methodology has strong potential to improve inspection efficiency, reduce economic losses, protect local agricultural ecosystems, and help ensure the quality and marketability of imported apples.

## Figures and Tables

**Table 1:** Primer sequence list.

Name	Primer Sequence	Size of Product (bp)
Botryosphaeria dothidea	ITS1:5′-TCCGTAGGTGAACCTGCGG-3′ ITS4:5′-TCCTCCGCTTATTGATATGC-3′	580
Monilinia fructicola	ITS1:5′-TATGCTCGCCAGAGGATAATT-3′ ITS4:5′-TGGGTTTTGGCAGAAGCACACT-3′	356
Cytospora mali	ITS1:5′-TCCGTAGGTGAACCTGCG-3′ ITS4:5′-TCCTCCGCTTATTGATAT-3′	560

**Table 2:** Hyperspectral Camera Specifications.

Main Technical Specifications	Parameters
Spectral Range	400–1000 nm
Spectral Resolution per Pixel	2.7 nm
Number of Spectral Bands	448
Spectral Resolution	5.5 nm
Number of Spatial Pixels	1024 px
Camera Frame Rate	320 FPS
Field of View	38°
Lens F-number	F/1.7
Data Interface	GigE Vision, CameraLink-
Connector	Industrial Ethernet OR CameraLink 26-pin, 0.5 MDR
Dimensions (L × W × H)	150 × 71 × 85 mm

**Table 3:** Comparison of Convolutional Neural Network Models on the Hyperspectral Dataset.

Model	Accuracy	Parameters
ShuffleNet	83.31%	5.42 M
MobileNet	87.23%	16.20 M
EfficientNet	86.72%	78.00 M
ViT	81.35%	210.00 M
ResNet	87.78%	97.26 M

**Table 4:** Hardware and Software Configuration Information.

Item	Parameters	Version
Hardware	CPU	Intel(R) Xeon(R) Gold 5317 CPU @ 3.00 GHz
	GPU	NVIDIA RTX 6000 Ada Generation 48 GB
	RAM	256 GB
Software	Python	3.12
	PyTorch	2.3.0
	CUDA	12.1
	CUDNN	8.9.1

**Table 5:** Model Hyperparameters.

Paramete	Value
Number of Workers	12
Batch Size	32
Epoch	100
Learning Rate	1 × 10^−4^
Optimizer	AdamW
LR Scheduler	CosineAnnealing

**Table 6:** Confusion Matrix Method.

Actual Class/Predicted Class	Predicted Positive	Predicted Negative
Actual Positive	TP	FN
Actual Negative	FP	TN

**Table 7:** Ablation Study Table.

Model	Baseline	channels	CBAM	Optimization	Accuracy
A	✔				87.78%
B	✔	✔			92.13%
C	✔	✔	✔		95.11%
D	✔	✔	✔	✔	95.51%

## Data Availability

The raw/processed data required to reproduce the above findings cannot be shared at this time, as the data also form part of an ongoing study.
